# Autophagy inhibition-mediated epithelial–mesenchymal transition augments local myofibroblast differentiation in pulmonary fibrosis

**DOI:** 10.1038/s41419-019-1820-x

**Published:** 2019-08-07

**Authors:** Charlotte Hill, Juanjuan Li, Dian Liu, Franco Conforti, Christopher J. Brereton, Liudi Yao, Yilu Zhou, Aiman Alzetani, Serena J. Chee, Ben G. Marshall, Sophie V. Fletcher, David Hancock, Christian H. Ottensmeier, Andrew J. Steele, Julian Downward, Luca Richeldi, Xin Lu, Donna E. Davies, Mark G. Jones, Yihua Wang

**Affiliations:** 1 Biological Sciences, Faculty of Environmental and Life Sciences, University of Southampton, Southampton, SO17 1BJ UK; 2 Department of Oncology, Tongji Hospital, Tongji Medical College, Huazhong University of Science and Technology, Wuhan, China; 3 Clinical and Experimental Sciences, Faculty of Medicine, University of Southampton, Southampton, SO16 6YD UK; 4 NIHR Southampton Biomedical Research Centre, University Hospital Southampton, Southampton, SO16 6YD UK; 5 University Hospital Southampton, Southampton, SO16 6YD UK; 6 Cancer Sciences Unit, University of Southampton, Somers Building, Southampton General Hospital, Southampton, SO16 6YD UK; 7 Oncogene Biology, The Francis Crick Institute, London, NW1 1AT UK; 8 Unità Operativa Complessa di Pneumologia, Università Cattolica del Sacro Cuore, Fondazione Policlinico A Gemelli IRCCS, Rome, Italy; 9 Ludwig Institute for Cancer Research, Nuffield Department of Clinical Medicine, University of Oxford, Oxford, OX3 7DQ UK; 10 Institute for Life Sciences, University of Southampton, Southampton, SO17 1BJ UK

**Keywords:** Macroautophagy, Respiratory tract diseases

## Abstract

Idiopathic pulmonary fibrosis (IPF), the prototypic progressive fibrotic interstitial lung disease, is thought to be a consequence of repetitive micro-injuries to an ageing, susceptible alveolar epithelium. Ageing is a risk factor for IPF and incidence has been demonstrated to increase with age. Decreased (macro)autophagy with age has been reported extensively in a variety of systems and diseases, including IPF. However, it is undetermined whether the role of faulty autophagy is causal or coincidental in the context of IPF. Here, we report that in alveolar epithelial cells inhibition of autophagy promotes epithelial–mesenchymal transition (EMT), a process implicated in embryonic development, wound healing, cancer metastasis and fibrosis. We further demonstrate that this is attained, at least in part, by increased p62/SQSTM1 expression that promotes p65/RELA mediated-transactivation of an EMT transcription factor, Snail2 (*SNAI2*), which not only controls EMT but also regulates the production of locally acting profibrogenic mediators. Our data suggest that reduced autophagy induces EMT of alveolar epithelial cells and can contribute to fibrosis via aberrant epithelial–fibroblast crosstalk.

## Introduction

Idiopathic pulmonary fibrosis (IPF) is the most common type of chronic, progressive fibrotic interstitial lung disease. It occurs with comparable incidence to that of stomach, brain and testicular cancer^1^, and the median survival of patients with IPF is only 3 years^2,3^. IPF is characterised by aberrant extracellular matrix (ECM) deposition, which leads to decreased lung compliance, disrupted gas-exchange and ultimately respiratory failure and death. As approved therapies only slow disease progression there is significant unmet medical need.

Evidence suggests that interacting genetic and environmental factors are crucial for the development of IPF, with repetitive injuries to aged alveolar epithelium thought to play an important role^2^. Ageing is a risk factor for IPF, with disease incidence increasing with age^3^. Decreased (macro)autophagy with age has been reported extensively in a variety of systems and diseases^4^, including IPF^5–9^. Autophagy is a tightly controlled, evolutionarily conserved process for the lysosomal degradation of cytoplasmic cargo, long-lived proteins and organelles. Growing evidence suggests that autophagy acts as an adaptive response to facilitate cell survival and limit cell death following exposure to stressful stimuli^10^.

Tissues from IPF patients are characterised by defective autophagy responses^5–8^, although it is unclear whether faulty autophagy exerts an effect directly, or if this change is merely coincident with IPF. Loss of *autophagy-related protein 7 (ATG7)*, a ubiquitin-activating enzyme that is essential for autophagy, has been demonstrated both in vivo and in vitro to induce endothelial-to-mesenchymal transition (EndMT). Autophagy inhibition, by loss of *ATG7*, has been associated with changes in endothelial cell (EC) architecture, reduced endothelial markers and increased mesenchymal markers, whilst EC-specific *Atg7* knockout mice had increased susceptibility to bleomycin induced-fibrosis^11^. Furthermore, inhibition of autophagy is sufficient to induce acceleration of both bronchial epithelial cell senescence and differentiation of lung fibroblasts into myofibroblasts^8^. Here, we report that autophagy inhibition promotes epithelial–mesenchymal transition (EMT) in alveolar epithelial cells. EMT is a reversible biological process where epithelial cells gain migratory and invasive abilities through loss of cell polarity and cadherin-mediated cell–cell adhesion; this process has been implicated in embryonic development, wound healing, cancer metastasis and organ fibrosis^12^. Specific transcription factors, EMT transcription factors (EMT-TFs), including Snail, ZEB and TWIST are able to promote the repression of epithelial features and induction of mesenchymal features^13,14^. We show that promotion of EMT by autophagy inhibition in alveolar epithelial cells is attained, at least in part, by increased p62/SQSTM1 expression that induces p65/RELA mediated-transactivation of Snail2 (*SNAI2*), which not only controls EMT but also regulates the production of locally acting profibrogenic mediators. This suggests that autophagy inhibition-induced EMT of alveolar epithelial cells contributes to fibrosis not only by affecting the epithelial phenotype but also via aberrant epithelial–fibroblast crosstalk.

## Results

### Autophagic activity is downregulated in IPF lungs

**Fig. 1:**
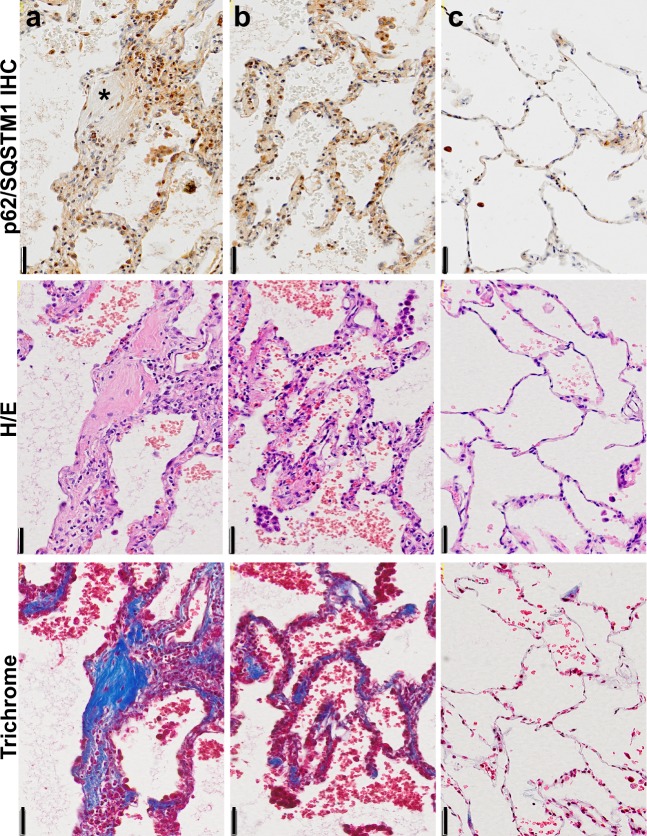
p62/SQSTM1 is highly expressed in IPF fibroblastic foci and epithelial cells of thickened alveoli septae where collagen deposition in the interstitium is also evident. Serial sections of IPF (fibroblastic foci in (**a**), and epithelial cells of thickened alveoli septae in (**b**)) or control lung tissue (**c**) were stained for p62/SQSTM1 (top panel), with H&E (middle panel) or Masson’s trichrome stain (bottom panel, collagen shown in blue). *n* = 3. *a fibroblastic focus. Scale bars: 50 μm

Defective autophagy responses in tissues from IPF patients have been reported previously^6,8^. Similarly, we observed evidence of autophagy inhibition as determined by p62/SQSTM1 accumulation in lung epithelial cells in IPF samples (Fig. 1; Supplementary Fig. S1a). p62/SQSTM1 is a key protein involved in autophagy that recognises cellular waste, which is then sequestered and degraded by autophagy^15^. A strong staining for p62/SQSTM1 was detected in IPF tissue within epithelial cells of thickened alveoli septae where collagen deposition in the interstitium was demonstrated (Fig. 1b; Supplementary Fig. S1a), as well as within fibroblastic foci (Fig. 1a). In contrast, there was weak p62/SQSTM1 expression and little collagen deposition detected in alveoli of control lung tissue (Fig. 1c). Using a publicly available dataset^16^, the mRNA level of *SQSTM1* (p62) in IPF epithelial cells was evaluated. It was found that *SQSTM1* (p62) mRNA expression level was reduced in IPF lungs (Supplementary Fig. S1b) whilst its protein level as assessed by immunohistochemistry was increased in IPF (Fig. 1b; Supplementary Fig. S1a). Given that p62/SQSTM1 protein levels are predominantly regulated by autophagic activity^17^, these results suggest that autophagic activity is downregulated in IPF epithelial cells.

### Alteration of autophagic activity affects cellular plasticity of alveolar epithelial cells

**Fig. 2:**
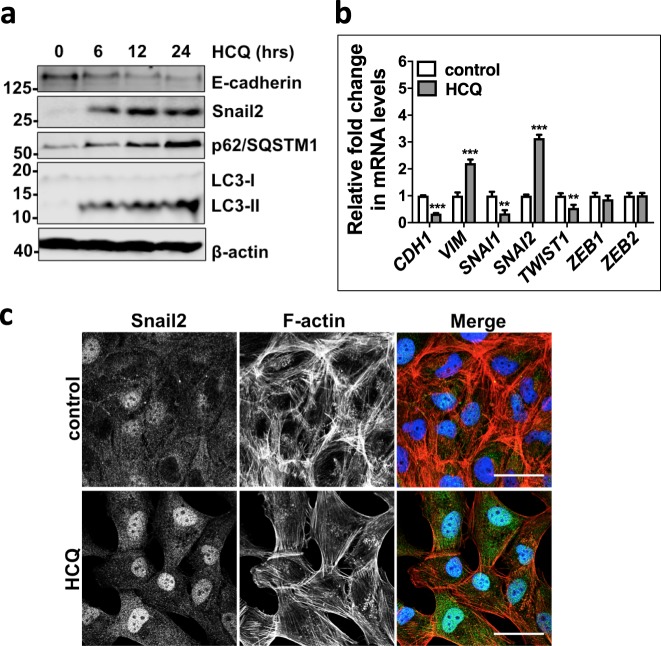
Autophagic activity altered by chemicals affects cellular plasticity of ATII cells. **a** Protein expression of E-Cadherin, Snail2, p62/SQSTM1 and LC3 in ATII cells treated with HCQ (25 μM) at indicated times. β-actin was used as a loading control. **b** Fold change in mRNA levels of *CDH1* (E-cadherin), *VIM* (Vimentin), *SNAI1* (Snail1), *SNAI2* (Snail2), *TWIST1*, *ZEB1* and *ZEB2* in ATII cells cultured in the absence or presence of HCQ (25 μM) for 24 hours. GAPDH-normalised mRNA levels in control cells were used to set the baseline value at unity. Data are mean ± s.d. *n* = 3 samples per group. ***P < 0.001. **c** Immunofluorescence staining of Snail2 (green) and F-actin (red) in ATII cells cultured in the absence or presence of HCQ (25 μM) for 24 h. Rhodamine-phalloidin was used to stain F-actin. DAPI (blue) was used to stain nuclei. Scale bar: 40 μm

To investigate its role in alveolar epithelial cells, autophagic activity was first modified using chemical inhibitors. We treated human alveolar epithelial type II (ATII) cells^18–20^ with an autophagy inhibitor, hydroxychloroquine (HCQ), which is a lysosomotropic autophagy inhibitor. HCQ treatment of ATII cells inhibited autophagic activity, demonstrated by the accumulation of p62/SQSTM1 (Fig. 2a), inhibition of lysosomal degradation by HCQ also caused accumulation of LC3-II (Fig. 2a). Autophagy inhibition with HCQ resulted in a downregulation of E-cadherin in a time-dependent manner (Fig. 2a). Morphology changes were also observed following HCQ treatment with ATII cells developing an elongated mesenchymal cell phenotype; this was accompanied by actin cytoskeleton reorganisation demonstrated using filamentous actin (F-actin) staining with Phalloidin (Fig. 2c), suggesting induction of a cellular reprogramming process, EMT. A group of transcription factors, termed EMT-TFs, are capable of inducing EMT, these include Snail1 (*SNAI1*), Snail2 (*SNAI2*), TWIST1, ZEB1 and ZEB2^13^, which act by binding E-boxes in the proximal promoter of the *CDH1* (E-cadherin) gene to repress its expression. We thus investigated the impact of HCQ treatment on the mRNA expression levels of E-cadherin (*CDH1*) and EMT-TFs in ATII cells. Following HCQ treatment of ATII cells, we observed a downregulation of E-cadherin (*CDH1*), increased levels of a mesenchymal marker Vimentin (*VIM*) and an EMT-TF Snail2 (*SNAI2*), but not others (Fig. 2b).

To confirm our observations with HCQ, we treated ATII cells with another autophagy inhibitor, Bafilomycin-A1 (Baf-A1), which inhibits autophagy by disrupting vesicular acidification leading to an accumulation of mature, undigested autophagosomes^21^. Autophagy inhibition by Baf-A1 treatment of ATII cells resulted in a similar induction of EMT, evidenced by a reduction in E-cadherin (Supplementary Fig. S2a), an increase of Vimentin (*VIM*) (Supplementary Fig. S2b), and an upregulation of EMT-TF Snail2 (*SNAI2*) (Supplementary Fig. S2b). Morphology changes were observed following Baf-A1 treatment of ATII cells, with cells developing invasive phenotype when cultured in Matrigel (Supplementary Fig. S2c).

**Fig. 3:**
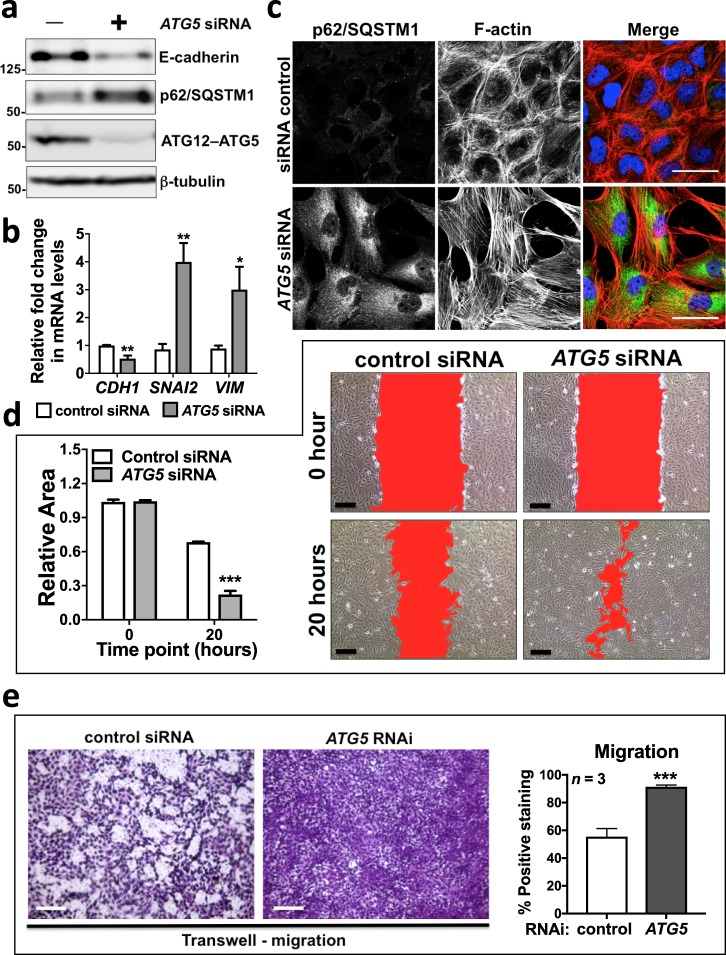
Autophagy inhibition by *ATG5* depletion induces EMT and cell migration in ATII cells. **a** Protein expression of E-cadherin, p62/SQSTM and ATG12–ATG5 in ATII cells transfected with indicated siRNAs. β-tubulin was used as a loading control. **b** Fold change in the mRNA levels of *CDH1* (E-cadherin), *SNAI2* (Snail2) and *VIM* (Vimentin) in ATII cells transfected with indicated siRNAs. GAPDH-normalised mRNA levels in control cells were used to set the baseline value at unity. Data are mean ± s.d. *n* = 3 samples per group. **P < 0.01. **c** Immunofluorescence staining of p62/SQSTM (green) and F-actin (red) in ATII cells transfected with control or *ATG5* siRNAs. Rhodamine-phalloidin was used to stain F-actin. DAPI (blue) was used to stain nuclei. Scale bar: 40 μm. **d** Scratch wound assay of ATII cells transfected with control or *ATG5* siRNAs. Representative images of ATII cells with the indicated treatments at time 0 or 20 h after the scratch wound. Wounds have been artificially coloured red to aid visualisation. Scale bar: 200 μm. The graph shows the area of a wound evaluated with ImageJ, and data are mean ± s.d. *n* = 3. ***P < 0.001. **e** Transwell migration assays in control or *ATG5*-depleted ATII cells. Cells were stained with crystal violet. Scale bar: 100 μm. Data are mean ± s.d. *n* = 3. ***P *<* 0.001

We further used small interfering RNAs (siRNAs) against *autophagy-related protein 5* (*ATG5*) to study the effects of inhibition of autophagic activity in ATII cells. ATG5, a ubiquitin-protein ligase (E3)-like enzyme, is essential for autophagy due to its role in autophagosome elongation. *ATG5* siRNA-transfection of ATII cells inhibited autophagic activity, evidenced by the accumulation of p62/SQSTM1 (Fig. 3a, c). As a result, *ATG5* depletion led to a downregulation of E-cadherin at both the mRNA and protein levels (Fig. 3a, b), an increase in *VIM* (Vimentin) at mRNA level (Fig. 3b), and a rearrangement of the F-actin cytoskeleton (Fig. 3c). Similar results were observed in A549 alveolar epithelial cells where autophagy inhibition by *ATG5* depletion down regulated E-cadherin levels and increased Snail2 (*SNAI2*) expression (Supplementary Fig. S3a–c).

In a wound healing context, the EMT process allows epithelial cells to adopt a more migratory mesenchymal phenotype in order to spread rapidly and cover the wounded area^22^. Therefore, a wound scratch assay was utilised to test whether *ATG5* depletion using *ATG5* siRNA was sufficient to augment cell mobility. This showed that 20 hours after creating the scratch wound, *ATG5* siRNA-transfected ATII cells had more completely repaired the wound compared to control siRNA-transfected cells (Fig. 3d; *P* < 0.001). In addition, we found in both the Transwell migration (Fig. 3e) and Matrigel invasion assays (Supplementary Fig. S3d), knockdown of *ATG5* promoted cell migration and invasion respectively.

Having examined the effects of autophagy inhibition in ATII cells, we next evaluated the effect of autophagy induction using Rapamycin, which inhibits mammalian Target Of Rapamycin (mTOR)^23^. As predicted, following Rapamycin treatment of ATII cells, phosphorylation levels of mTOR were reduced (Supplementary Fig. S4a), and autophagy activity was induced, indicated by a downregulation of p62/SQSTM1 and an increase in LC3-II (Supplementary Fig. S4a). We also found an increase in *CDH1* (E-cadherin), and reductions in *VIM* (Vimentin) and *SNAI2* (Snail2) mRNA levels in ATII cells treated with Rapamycin (Supplementary Fig. S4b), and these effects were mainly via the induction of autophagy, since knockdown of *ATG5* completely abolished the changes (Supplementary Fig. S4b).

Together, our results demonstrate that autophagic activity is an important regulator of cellular plasticity in alveolar epithelial cells.

### Autophagy inhibition induces EMT via p62/SQSMT1-NFκB-Snail2 pathway in alveolar epithelial cells

**Fig. 4:**
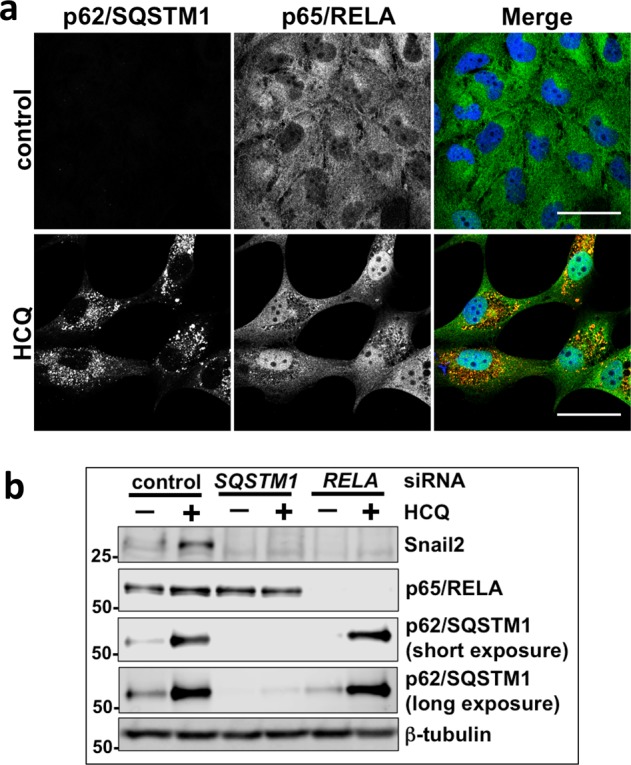
Autophagy inhibition induces EMT via p62/SQSTM1-NF-κB-Snail2 pathway in ATII cells. **a** Immunofluorescence staining of p62/SQSTM1 (red) and p65/RELA (green) in ATII cells treated with hydroxychloroquine (HCQ, 25 μM) for 24 h. DAPI (blue) was used to stain nuclei. Scale bar: 40 μm. **b** Protein expression of Snail2, p65/RELA and p62/SQSTM1 in ATII cells transfected with the indicated siRNAs followed by treatment of HCQ (25 μM) for 24 h. For protein expression of p62/SQSTM1, both short and long exposures (respectively) are shown. β-tubulin was used as a loading control

We recently reported that in colorectal and pancreatic cancer cells, inhibition of autophagy induces EMT via p62/SQSMT1-NFκB pathway^24^. Therefore, we investigated if this pathway is involved in initiating autophagy inhibition-induced EMT in ATII cells. Comparison of control and HCQ-treated cells revealed that p65/RELA accumulated in the nuclei of treated cells (Fig. 4a), indicating that the NF-κB pathway is activated in ATII cells when autophagy is inhibited. Functionally, p62/*SQSTM1* or p65/*RELA* knockdown abolished the increase in Snail2 following autophagy inhibition by HCQ treatment in ATII cells (Fig. 4b). Similar results were observed in A549 cells (Supplementary Fig. S5). While knockdown of *ATG5* induced EMT, as evidenced by a reduction in *CDH1* (E-cadherin) mRNA levels and increased *SNAI2* (Snail2) mRNA levels, subsequent depletion of *RELA* (p65) or *SQSTM1* (p62) (Supplementary Fig. S5a) partially or completely abolished the increase in *SNAI2* (Snail2), and restored *CDH1* (E-cadherin) expression (Supplementary Fig. S5b). These data suggest that autophagy inhibition in alveolar epithelial cells promotes EMT via the p62/SQSMT1-NFκB-Snail2 pathway.

### ATII cells undergoing autophagy inhibition-induced EMT induce fibroblast activation via Snail2-regulated paracrine signalling

**Fig. 5:**
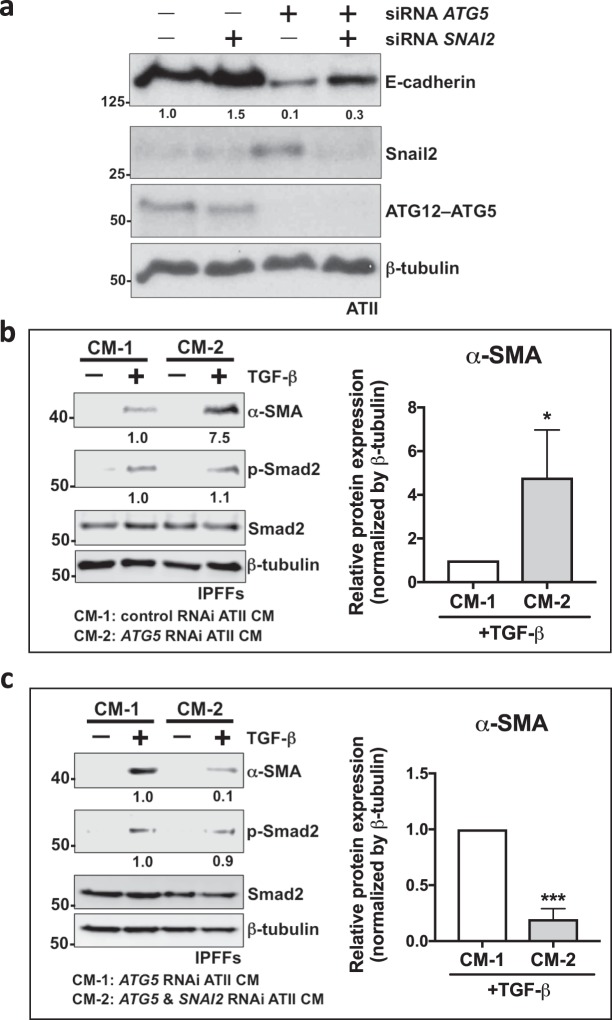
ATII cells undergoing autophagy inhibition-induced EMT induce fibroblast activation via Snail2-regulated paracrine signalling. **a** Protein expression of E-cadherin, ATG12–ATG5 and Snail2 in ATII cells transfected with indicated siRNAs. β-tubulin was used as a loading control. **b, c** Protein expression of α-SMA, Smad2 and phospho-Smad2 (p-Smad2) in IPFFs with indicated treatments. β-tubulin was used as a loading control. Scores under the bands are relative levels when compared with indicated controls (1.0). The graphs show relative α-SMA protein levels evaluated with ImageJ, and data are mean ± s.d. *n* = 3. *P < 0.05. ***P < 0.001

Given that autophagy inhibition is able to induce EMT in ATII cells, we wanted to determine the role of these cells in the context of fibrosis, and whether they were contributing to the fibroblast population directly. Comparison of ECM components in control or *ATG5* siRNA ATII cells demonstrated that ATII cells after autophagy inhibition-induced EMT did not express significantly more ECM genes (Supplementary Fig. S6). This suggested ECM production in IPF lung tissue (Fig. 1) was unlikely due directly to EMT but rather epithelial cells exhibiting an indirect effect on fibrogenesis. Therefore, we investigated whether *ATG5*-depleted ATII cells could produce secreted factors that activate fibroblasts. We treated primary human lung fibroblasts from IPF patients (IPF fibroblasts, IPFFs) with conditioned media (CM) from ATII cells transfected with control or *ATG5* siRNA (Fig. 5a) without or with addition of transforming growth factor-β (TGF-β), and assessed levels of α-smooth muscle actin (α-SMA), a marker of myofibroblast differentiation. CM from ATII cells transfected with *ATG5* siRNA without TGF-β had minimal effect on the activation of fibroblasts (Fig. 5b). However in IPFFs, CM from *ATG5*-depleted ATII cells together with TGF-β achieved a synergistic effect in activating fibroblasts as assessed by α-SMA protein levels (Fig. 5b). Notably, levels of phospho-Smad2 (p-Smad2) did not significantly change between different CM with TGF-β treatment, suggesting α-SMA increase may be Smad2-independent (Fig. 5b). Given the importance of Snail2 (*SNAI2*) in mediating EMT by autophagy inhibition (Fig. 5a), we hypothesised that Snail2 (*SNAI2*) may facilitate crosstalk by mediating paracrine signalling from ATII cells undergoing autophagy inhibition-induced EMT. Snail2 (*SNAI2*) depletion (Fig. 5a) in ATII cells eliminated the effects of CM from *ATG5*-depleted ATII cells on activation of fibroblasts in the presence of TGF-β (Fig. 5c), highlighting the importance of Snail2 (*SNAI2*) as a key regulator of paracrine signalling between alveolar cells and fibroblasts in IPF under conditions where autophagy is defective.

## Discussion

Fibrotic diseases are poorly characterised and lack effective treatment^25^, with fibrosis presenting as a pathological feature in a range of diseases affecting many organs^25,26^. A rise in the incidence of some fibrotic diseases^1,3^ and with fibrogenesis being accepted as a major cause of death^27^; it is evident that they are becoming an increasing healthcare burden, requiring further research to elucidate underlying mechanisms which drive fibrogenesis. IPF is an aging-associated chronic, interstitial lung disease^2,28–34^. Treatment options for patients are limited^35^ and the underlying mechanisms for fibrosis are still debated^36^. EMT has been suggested to have a direct role in IPF, with studies showing co-localisation of epithelial and mesenchymal markers^37–41^, and laser capture micro-dissection isolated RNA from epithelial cells in IPF lungs confirmed expression of mesenchymal markers^42^. However, lineage tracing studies found the number of fibroblasts derived from epithelial cells to be small^43,44^, and these cells were not found to co-localise with α-SMA suggesting they did not transition to myofibroblasts^45,46^. It also been proposed in a number of systems that epithelial cells that have undergone EMT may secrete a range of fibrogenic growth factors and cytokines, with impaired epithelial repair leading to aberrant epithelial–mesenchymal communication contributing to the recruitment and activation of myofibroblasts^18,47,48^. Here, we show that autophagy inhibition in ATII cells can induce EMT, and this contributes to fibrosis via aberrant epithelial–fibroblast crosstalk, rather than as a direct contribution to the fibroblast population.

Autophagy is a tightly controlled, evolutionarily conserved biological process where long-lived proteins and damaged organelles are degraded. Manipulation of autophagy is now being utilised as a therapeutic approach in a number of fields, such as, neurodegenerative diseases and cancer^49,50^. On the other hand, decreased autophagic activity has been reported in many human diseases^51^, including IPF^5–8^. In IPF, ageing is a main risk factor in its development with patients under the age of 50 being rare^29^. Ageing has been linked to reduced autophagy in many contexts, including IPF^5,52^. Consistent with these findings^6,8^ we demonstrated strong immunostaining for p62/SQSTM1 in IPF epithelial cells of thickened alveolar septae whilst identifying only very weak signals in the control lung. We found significantly lower levels of *SQSTM1* (p62) mRNA in IPF epithelial cells compared to control epithelial cells, which indicated that the increase in p62/SQSTM1 protein levels was due to reduced autophagic activity. Taken with previous findings^6,8^, these results indicate that autophagic activity is downregulated in IPF epithelial cells.

**Fig. 6:**
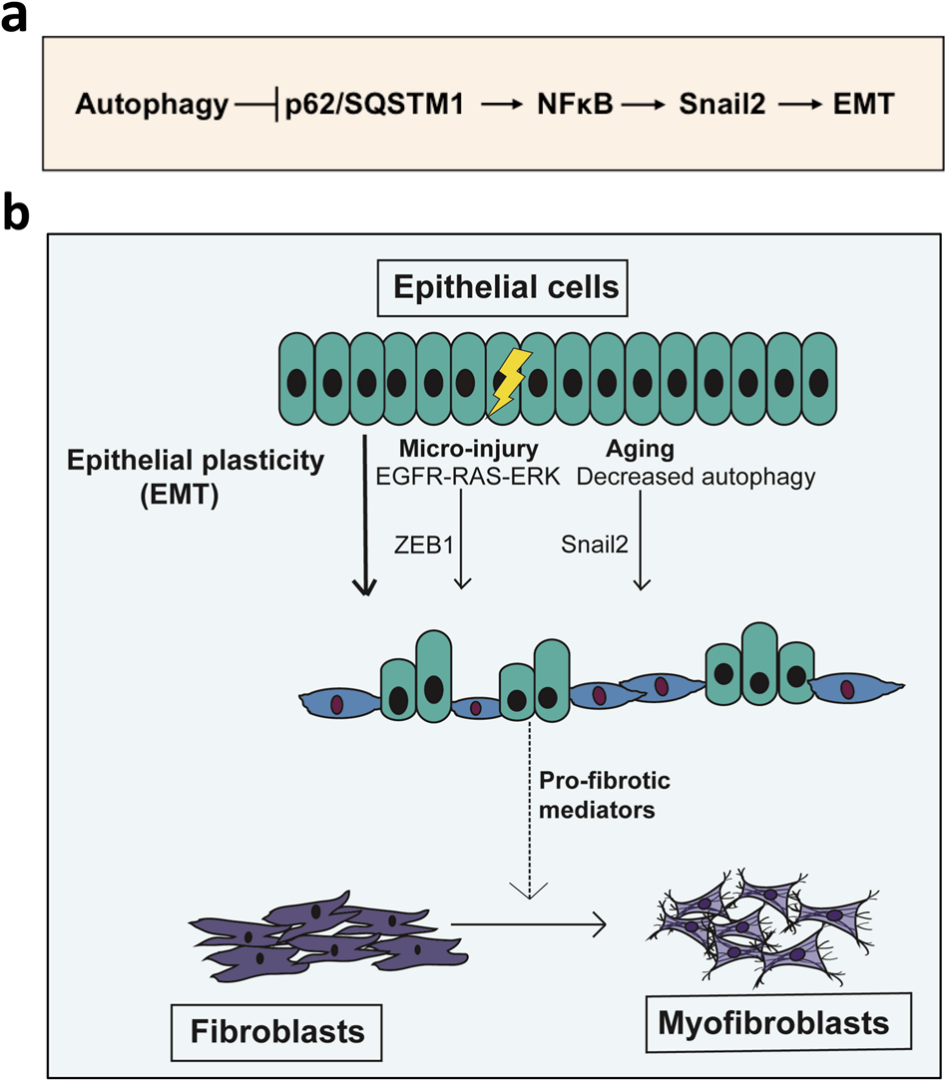
Autophagy inhibition-mediated epithelial–mesenchymal transition augments local myofibroblast differentiation in pulmonary fibrosis. Diagrams showing autophagy inhibition induces EMT via p62/SQSMT1-NFκB-Snail2 pathway in alveolar epithelial cells (**a**) and local micro-injuries to ageing alveolar epithelium causes persistent activation of alveolar epithelial cells, which secrete numerous profibrogenic factors, driving local myofibroblast differentiation (**b**)

Whilst the concept of EMT is well established in the context of embryonic development, it also plays a role in in wound healing, cancer metastasis and fibrosis^12^. EMT requires a complex orchestration of multiple signalling pathways, including TGF-β, fibroblast growth factor, Wnt/β-catenin, epidermal growth factor (EGF) and others. Loss of E-cadherin is considered to be a fundamental event in EMT. Snail1/2, ZEB1/2 and some basic helix-loop-helix factors are potent repressors of E-cadherin expression^53,54^. The role of EMT in cancer is detrimental whereas in wound healing, EMT as a response to injury can be beneficial, however, if the wound healing process is exaggerated it may lead to fibrosis. The interplay between autophagy and EMT have been reported in other disease contexts^55–57^, with a recent study demonstrating inhibition of autophagy induces EMT via p62/SQSMT1-NFκB/RELA pathway in *RAS*-mutated cancer cells^24^. This is mainly via upregulation of ZEB1 and to a lesser extent via Snail2^24^. In contrast, in lung alveolar epithelial cells, autophagy inhibition induces EMT in the absence of RAS activation and is exclusively via up-regulation of Snail2 (Fig. 6a), indicating this effect is likely dependent on the cellular context. The importance of Snail2 in IPF has been demonstrated with Snail2 being upregulated in IPF lung epithelial cells, but not Snail1 or TWIST1, compared to control epithelial cells^16,18^. Jayachandran et al.^54^ reported that Snail2-mediated EMT may contribute to the fibroblast pool. However, in this study, we found ATII cells undergoing autophagy inhibition-induced EMT induce fibroblast activation via Snail2-regulated paracrine signalling.

In IPF it was recently reported that micro-injuries could lead to the activation of the EGFR-RAS-ERK pathway, and ZEB1 was demonstrated to mediate paracrine signalling^18^. Similarly in renal fibrosis, tubular epithelial cells are able to promote myofibroblast differentiation and fibrogenesis without directly contributing to the fibroblast population by relaying signals to the interstitium. For example, in a murine model, damage-mediated Snail1 reactivation-induced partial EMT and resulted in renal fibrosis, but these cells did not contribute to myofibroblast or interstitial cell population^47^. Conversely, EMT was inhibited and interstitial fibrosis attenuated upon conditional deletion of *Snail1* or *Twist1*^48^.

Taken together, we believe that repetitive local micro-injuries to ageing alveolar epithelium cause persistent activation of alveolar epithelial cells, which secrete numerous profibrogenic factors, driving local myofibroblast differentiation (Fig. 6b). Thus, targeting of EMT inducers might have therapeutic potential in fibrotic conditions, with such therapies currently undergoing development in the context of malignancy^58,59^.

## Methods

### Lung tissue sampling

All human lung experiments were approved by the Southampton and South West Hampshire and the Mid and South Buckinghamshire Local Research Ethics Committees, and all subjects gave written informed consent. Clinically indicated IPF lung biopsy tissue samples and non-fibrotic control tissue samples (macroscopically normal lung sampled remote from a cancer site) were assessed as surplus to clinical diagnostic requirements. All IPF samples were from patients subsequently receiving a multidisciplinary diagnosis of IPF according to international consensus guidelines^29^.

### Cell culture, reagents and transfections

Primary parenchymal lung fibroblast cultures were established from IPF tissues as described previously^60,61^. Fibroblasts were cultured in Dulbecco’s Modified Eagle’s Medium (DMEM) supplemented with 10% foetal bovine serum (FBS), 50 units/ml penicillin, 50 μg/ml streptomycin, 2 mM l-glutamine, 1 mM sodium pyruvate and 1× non-essential amino acids (DMEM/FBS) (all from Life Technologies). ATII cells^18–20^ were cultured in DCCM-1 (Biological Industries Ltd.) supplemented with 10% NBCS (Life Technologies), 1% penicillin, 1% streptomycin and 1% l-glutamine (all from Sigma Aldrich). A549 cells were cultured in DMEM (Fisher Scientific UK, 11594446) supplemented with 10% FBS (Invitrogen) and antibiotics. All cells were kept at 37 °C and 5% CO_2_. For 3D culture, ATII cells were cultured as previously described^62^ in Matrigel (BD Biosciences). Bafilomycin-A1 (Baf-A1) was from Enzo Life Sciences. HCQ and Rapamycin were from Sigma Aldrich. No mycoplasma contamination was detected in the cell lines used.

siRNA oligos against *ATG5* (MU-004374-04-0002), *SQSTM1* (p62) (MU-010230-00-0002), *SNAI2* (Snail2) (MU-017386-00-0002) and *RELA* (p65) (MU-003533-02-0002) were purchased from Dharmacon. Sequences are available from Dharmacon, or on request. As a negative control we used siGENOME RISC-Free siRNA (Dharmacon, D-001220-01). ATII and A549 cells were transfected with the indicated siRNA oligos at a final concentration of 35 nM using DharmaFECT 2 reagent (Dharmacon).

### Western blot analysis

Western blot analysis was performed with lysates from cells with urea buffer (8 M Urea, 1 M Thiourea, 0.5% CHAPS, 50 mM DTT, and 24 mM Spermine). Primary antibodies were from: Santa Cruz (β-actin, sc-47778; E-cadherin, sc-21791; Snail2, sc-10436), Abcam (β-tubulin, ab6046), Cell Signalling Technology (α-SMA, 14968; Snail2, 9585; Smad2, 5339; Phospho-Smad2, 3104; β-tubulin, 86298; LC3, 2775; p62/SQSTM1, 5114; p65/RELA, 8242; ATG5, 2630; mTOR, 2972; Phospho-mTOR Ser2448, 5536), and BD Transduction Laboratories (E-cadherin, 610405). Signals were detected using an ECL detection system (GE Healthcare) or Odyssey imaging system (LI-COR), and evaluated by ImageJ 1.42q software (National Institutes of Health).

### qRT-PCR

Total RNA was isolated using RNeasy mini kit (Qiagen) according to manufacturer’s instructions and quantified using a Nanodrop Spectophotometer 2000c (Thermo Fisher Scientific). Real-time quantitative RT-PCR was carried out using gene-specific primers (QuantiTect Primer Assays, Qiagen) for *CDH1* (E-cadherin) (QT00080143), *SNAI1* (Snail1) (QT00010010), *SNAI2* (Snail2) (QT00044128), *ZEB1* (QT00008555), *ZEB2* (QT00008554), *TWIST1* (QT00011956), *COL1A1* (QT00037793), *ACTA2* (α-SMA) (QT00088102), *COL3A1* (QT00058233)*, FN1* (QT00038024*), VIM* (QT00095795)*, GAPDH* (QT01192646) or *ACTB* (β-actin) (QT01680476) with QuantiNova SYBR Green RT-PCR kits (Qiagen). Relative transcript levels of target genes were normalised to *GAPDH* or *ACTB* (β-actin).

### Immunofluorescence microscopy

Cells were fixed in 4% phosphate-buffered saline (PBS)–paraformaldehyde for 15 min, incubated in 0.1% Triton X-100 for 5 min on ice, then in 0.2% fish skin gelatine in PBS for 1 h and stained for 1 h with an anti-E-cadherin (1:100, BD Biosciences, 610182) or anti-p62/SQSTM1 (1:100, BD Biosciences, 610833) or anti-Snail2 (1:100, Cell Signalling Technology, 9585), p65/RELA (1:100, Cell Signalling Technology, 8242). Protein expression was detected using Alexa Fluor (1:400, Molecular Probes) for 20 min. DAPI or TO-PRO-3 (Invitrogen) was used to stain nuclei (1:1000). Rhodamine phalloidin was used to visualise filamentous actin (F-actin) (Molecular Probes). For immunofluorescence staining of 3D cultures from ATII cells, spheres were fixed with 4% PBS–paraformaldehyde for 40 min, permeabilised in 0.5% Triton X-100 for 10 min on ice and stained with rhodamine phalloidin for 1 h at room temperature. Spheres were counterstained with DAPI. Samples were observed using a confocal microscope system (Leica SP8). Acquired images were analysed using Photoshop (Adobe Systems) according to the guidelines of the journal.

### Wound-healing migration assay

The wound-healing migration assays were done in conjunction with siRNA transfections in ATII cells. Seventy-two hours after siRNA transfections, confluent monolayers of cells were wounded with a p20 pipette tip (time 0). Phase-contrast images were taken using an Olympus inverted microscope at time 0 or 20 h after the scratch wound, invasive cells were evaluated by ImageJ software (National Institutes of Health).

### Transwell migration and Matrigel invasion assay

For the Transwell migration assay, Transwell membranes (8-μm pore size, 6.5-mm diameter; Corning Costar, 3422) were used. The bottom chambers of the Transwell were filled with migration-inducing medium (with 50% FBS). The top chambers were seeded with 1.5 × 10^5^ live serum-starved control or *ATG5*-depleted ATII cells per well. After 24 h, the filters were fixed with 4% paraformaldehyde for 10 min at room temperature; subsequently, the cells on the upper side of the membrane were scraped with a cotton swab. Similar inserts coated with Matrigel (Corning, 354480) were used to determine invasive potential in invasion assays. Filters were stained with crystal violet for light microscopy. Images were taken using an Olympus inverted microscope and migratory cells were evaluated by ImageJ 1.42q software (National Institutes of Health).

### Immunohistochemistry, haematoxylin and eosin (H/E) and tinctorial stains

Control or IPF lung tissues were fixed and embedded in paraffin wax; tissue sections (4 µm) were processed and stained as previously described^18^. Briefly, the tissue sections were de-waxed, rehydrated and incubated with 3% hydrogen peroxide in methanol for 10 min to block endogenous peroxidase activity. Sections were then blocked with normal goat serum and incubated at room temperature with a primary antibody against p62/SQSTM1 (1:100, Progen, GP62_C), followed by a biotinylated secondary antibody (1:500, Vector Laboratories Ltd., UK); antibody binding was detected using streptavidin-conjugated horse-radish peroxidase and visualised using DAB (DAKO) before counterstaining with Mayer’s Haematoxylin. For H/E stain, Shandon Varistain 24-4 automatic slide stainer (Thermo Fisher Scientific) was used. For tinctorial stain, Trichrome stain (Abcam ab150686) was used according to the manufacturers’ instructions. Images were acquired using an Olympus Dotslide Scanner VS110.

### Statistical analysis and repeatability of experiments

Each experiment was repeated at least twice. Unless otherwise noted, data are presented as mean and s.d., and a two-tailed, unpaired or paired Student’s *t* test was used to compare two groups for independent samples. *P* < 0.05 was considered statistically significant.

## Supplementary information

Supplementary Text.

Supplementary Figure S1.

Supplementary Figure S2.

Supplementary Figure S3.

Supplementary Figure S4.

Supplementary Figure S5.

Supplementary Figure S6.

## References

[1] Hutchinson J, Fogarty A, Hubbard R, McKeever T (2015). Global incidence and mortality of idiopathic pulmonary fibrosis: a systematic review. Eur. Respir. J..

[2] Richeldi L, Collard HR, Jones MG (2017). Idiopathic pulmonary fibrosis. Lancet.

[3] Raghu G, Weycker D, Edelsberg J, Bradford WZ, Oster G (2006). Incidence and prevalence of idiopathic pulmonary fibrosis. Am. J. Respir. Crit. Care Med..

[4] Martinez-Lopez N, Athonvarangkul D, Singh R (2015). Autophagy and aging. Adv. Exp. Med. Biol..

[5] Romero Y (2016). mTORC1 activation decreases autophagy in aging and idiopathic pulmonary fibrosis and contributes to apoptosis resistance in IPF fibroblasts. Aging Cell.

[6] Patel AS (2012). Autophagy in idiopathic pulmonary fibrosis. PLoS ONE.

[7] Rangarajan S (2016). Novel mechanisms for the antifibrotic action of nintedanib. Am. J. Respir. Cell Mol. Biol..

[8] Araya J (2013). Insufficient autophagy in idiopathic pulmonary fibrosis. Am. J. Physiol. Lung Cell Mol. Physiol..

[9] Ghavami S (2018). Autophagy and the unfolded protein response promote profibrotic effects of TGF-β _1_ in human lung fibroblasts. Am. J. Physiol. Cell Mol. Physiol..

[10] Bento CF (2016). Mammalian autophagy: how does it work?. Annu Rev. Biochem..

[11] Singh KK (2015). The essential autophagy gene ATG7 modulates organ fibrosis via regulation of endothelial-to-mesenchymal transition. J. Biol. Chem..

[12] Nieto A, Huang R, Jackson R, Thiery J (2016). EMT: 2016. Cell.

[13] Peinado H, Olmeda D, Cano A (2007). Snail, Zeb and bHLH factors in tumour progression: an alliance against the epithelial phenotype?. Nat. Rev. Cancer.

[14] Nieto MA (2011). The ins and outs of the epithelial to mesenchymal transition in health and disease. Annu Rev. Cell Dev. Biol..

[15] Rusten TE, Stenmark H (2010). p62, an autophagy hero or culprit?. Nat. Cell Biol..

[16] Xu Y (2017). Single-cell RNA sequencing identifies diverse roles of epithelial cells in idiopathic pulmonary fibrosis. JCI Insight.

[17] Moscat J, Diaz-Meco MT (2009). p62 at the crossroads of autophagy, apoptosis, and cancer. Cell.

[18] Yao Liudi, Conforti Franco, Hill Charlotte, Bell Joseph, Drawater Leena, Li Juanjuan, Liu Dian, Xiong Hua, Alzetani Aiman, Chee Serena J., Marshall Ben G., Fletcher Sophie V., Hancock David, Coldwell Mark, Yuan Xianglin, Ottensmeier Christian H., Downward Julian, Collins Jane E., Ewing Rob M., Richeldi Luca, Skipp Paul, Jones Mark G., Davies Donna E., Wang Yihua (2018). Paracrine signalling during ZEB1-mediated epithelial–mesenchymal transition augments local myofibroblast differentiation in lung fibrosis. Cell Death & Differentiation.

[19] Molina-Arcas M, Hancock DC, Sheridan C, Kumar MS, Downward J (2013). Coordinate direct input of both KRAS and IGF1 receptor to activation of PI3 kinase in *KRAS*-mutant lung cancer. Cancer Disco..

[20] Coelho MA (2017). Oncogenic RAS signaling promotes tumor immunoresistance by stabilizing PD-L1 mRNA. Immunity.

[21] DeVorkin, L. & Lum, J. J. In: Strategies to block autophagy in tumor cells. *Autophagy: Cancer, Other Pathologies, Inflammation, Immunity, Infection, and Aging*. (ed. Hayat, M.) 121–130 (Academic Press: New York, 2014).

[22] Thiery JP, Sleeman JP (2006). Complex networks orchestrate epithelial–mesenchymal transitions. Nat. Rev. Mol. Cell Biol..

[23] Ballou LM, Lin RZ (2008). Rapamycin and mTOR kinase inhibitors. J. Chem. Biol..

[24] Wang Yihua, Xiong Hua, Liu Dian, Hill Charlotte, Ertay Ayse, Li Juanjuan, Zou Yanmei, Miller Paul, White Eileen, Downward Julian, Goldin Robert D, Yuan Xianglin, Lu Xin (2019). Autophagy inhibition specifically promotes epithelial-mesenchymal transition and invasion in RAS-mutated cancer cells. Autophagy.

[25] Thannickal VJ, Zhou Y, Gaggar A, Duncan SR (2014). Fibrosis: ultimate and proximate causes. J. Clin. Invest..

[26] Wynn TA (2004). Fibrotic disease and the T(H)1/T(H)2 paradigm. Nat. Rev. Immunol..

[27] Wynn TA, Ramalingam TR (2012). Mechanisms of fibrosis: therapeutic translation for fibrotic disease. Nat. Med..

[28] Pardo A, Selman M (2016). Lung fibroblasts, aging, and idiopathic pulmonary fibrosis. Ann. Am. Thorac. Soc..

[29] Raghu G (2018). Diagnosis of idiopathic pulmonary fibrosis. An Official ATS/ERS/JRS/ALAT Clinical Practice Guideline. Am. J. Respir. Crit. Care Med..

[30] Minagawa S (2011). Accelerated epithelial cell senescence in IPF and the inhibitory role of SIRT6 in TGF-β-induced senescence of human bronchial epithelial cells. Am. J. Physiol. Cell Mol. Physiol..

[31] Tsakiri KD (2007). Adult-onset pulmonary fibrosis caused by mutations in telomerase. Proc. Natl Acad. Sci. USA.

[32] Alder JK (2008). Short telomeres are a risk factor for idiopathic pulmonary fibrosis. Proc. Natl Acad. Sci. USA.

[33] Bueno M (2015). PINK1 deficiency impairs mitochondrial homeostasis and promotes lung fibrosis. J. Clin. Invest..

[34] Fingerlin TE (2013). Genome-wide association study identifies multiple susceptibility loci for pulmonary fibrosis. Nat. Genet..

[35] Spagnolo P, Tzouvelekis A, Bonella F (2018). The management of patients with idiopathic pulmonary fibrosis. Front. Med.

[36] Hill C, Jones MG, Davies DE, Wang Y (2019). Epithelial-mesenchymal transition contributes to pulmonary fibrosis via aberrant epithelial/fibroblastic cross-talk. J. Lung Health Dis..

[37] Chilosi M (2017). Epithelial to mesenchymal transition-related proteins ZEB1, β-catenin and β-tubulin-III in idiopathic pulmonary fibrosis. Mod. Pathol..

[38] Park JS (2014). Clinical significance of mTOR, ZEB1, ROCK1 expression in lung tissues of pulmonary fibrosis patients. BMC Pulm. Med..

[39] Lomas NJ, Watts KL, Akram KM, Forsyth NR, Spiteri MA (2012). Idiopathic pulmonary fibrosis: immunohistochemical analysis provides fresh insights into lung tissue remodelling with implications for novel prognostic markers. Int J. Clin. Exp. Pathol..

[40] Willis BC (2005). Induction of epithelial-mesenchymal transition in alveolar epithelial cells by transforming growth factor-beta1: potential role in idiopathic pulmonary fibrosis. Am. J. Pathol..

[41] Harada T (2010). Epithelial–mesenchymal transition in human lungs with usual interstitial pneumonia: quantitative immunohistochemistry. Pathol. Int..

[42] Marmai C (2011). Alveolar epithelial cells express mesenchymal proteins in patients with idiopathic pulmonary fibrosis. AJP Lung Cell Mol. Physiol..

[43] Rock JR (2011). Multiple stromal populations contribute to pulmonary fibrosis without evidence for epithelial to mesenchymal transition. Proc. Natl Acad. Sci..

[44] Humphreys BD (2010). Fate tracing reveals the pericyte and not epithelial origin of myofibroblasts in kidney fibrosis. Am. J. Pathol..

[45] Tanjore H (2009). Contribution of epithelial-derived fibroblasts to bleomycin-induced lung fibrosis. Am. J. Respir. Crit. Care Med..

[46] Degryse AL (2011). TGFβ signaling in lung epithelium regulates bleomycin-induced alveolar injury and fibroblast recruitment. Am. J. Physiol. Lung Cell Mol. Physiol..

[47] Grande MT (2015). Snail1-induced partial epithelial-to-mesenchymal transition drives renal fibrosis in mice and can be targeted to reverse established disease. Nat. Med..

[48] Lovisa S (2015). Epithelial-to-mesenchymal transition induces cell cycle arrest and parenchymal damage in renal fibrosis. Nat. Med..

[49] Harris H, Rubinsztein DC (2012). Control of autophagy as a therapy for neurodegenerative disease. Nat. Rev. Neurol..

[50] White E (2012). Deconvoluting the context-dependent role for autophagy in cancer. Nat. Rev. Cancer.

[51] Saha S, Panigrahi DP, Patil S, Bhutia SK (2018). Autophagy in health and disease: a comprehensive review. Biomed. Pharm..

[52] Rubinsztein DC, Mariño G, Kroemer G (2011). Autophagy and aging. Cell.

[53] Lamouille S, Xu J, Derynck R (2014). Molecular mechanisms of epithelial–mesenchymal transition. Nat. Rev. Mol. Cell Biol..

[54] Jayachandran A (2009). SNAI transcription factors mediate epithelial-mesenchymal transition in lung fibrosis. Thorax.

[55] Catalano M (2015). Autophagy induction impairs migration and invasion by reversing EMT in glioblastoma cells. Mol. Oncol..

[56] Wei R (2019). FAT4 regulates the EMT and autophagy in colorectal cancer cells in part via the PI3K-AKT signaling axis. J. Exp. Clin. Cancer Res..

[57] Gugnoni M, Sancisi V, Manzotti G, Gandolfi G, Ciarrocchi A (2016). Autophagy and epithelial–mesenchymal transition: an intricate interplay in cancer. Cell Death Dis..

[58] Sakata J (2017). Inhibition of ZEB1 leads to inversion of metastatic characteristics and restoration of paclitaxel sensitivity of chronic chemoresistant ovarian carcinoma cells. Oncotarget.

[59] Kothari A, Mi Z, Zapf M, Kuo P (2014). Novel clinical therapeutics targeting the epithelial to mesenchymal transition. Clin. Transl. Med..

[60] Conforti F (2017). The histone deacetylase inhibitor, romidepsin, as a potential treatment for pulmonary fibrosis. Oncotarget.

[61] Jones MG (2018). Nanoscale dysregulation of collagen structure-function disrupts mechano-homeostasis and mediates pulmonary fibrosis. Elife.

[62] Yu W (2007). Formation of cysts by alveolar type II cells in three-dimensional culture reveals a novel mechanism for epithelial morphogenesis. Mol. Biol. Cell.

