# Supplementary material for: Autophagy inhibition-mediated epithelial–mesenchymal transition augments local myofibroblast differentiation in pulmonary fibrosis
Source: Cell Death Dis. 2019 Aug 7;10(8):591. doi: 10.1038/s41419-019-1820-x (PMC6685977; doi:10.1038/s41419-019-1820-x)
Supplement: Supplementary file 1 — Supplementary Text. [file 41419_2019_1820_MOESM1_ESM.docx]

**Autophagy inhibition-mediated epithelial mesenchymal transition augments local myofibroblast differentiation in pulmonary fibrosis**

**Supplementary Figure Legends**

**Supplementary Figure 1** The protein level, but not the mRNA level, of p62/SQSTM1 is up regulated in IPF epithelial cells. **(a)** Sections from IPF or control lung tissues were stained for p62/SQSTM1. Scale bars: 50 μm. **(b)** Decreased mRNA levels of *SQSTM1* (p62) in IPF epithelial cells are shown by an online LGEA Web Portal (<https://research.cchmc.org/pbge/lunggens/mainportal.html>).

**Supplementary Figure 2** Autophagy inhibition with Bafilomycin A1 (Baf-A1) induces EMT in ATII cells. (**a**) Protein expression of E-cadherin, p62/SQSTM1 and LC3 in ATII cells treated with Baf-A1 (10nM) for the indicated period. β-actin was used as a loading control. (**b**) Fold change in mRNA levels of *CDH1* (E-cadherin), *VIM* (Vimentin), *SNAI1* (Snail1), *SNAI2* (Snail2), *TWIST1*, *ZEB1* and *ZEB2* in ATII cells cultured in the absence or presence of Baf-A1 (10 nM) for 24 hrs. GAPDH-normalised mRNA levels in control cells were used to set the baseline value at unity. Data are mean ± s.d. *n* = 3 samples per group. ** *P* < 0.01. *** *P* < 0.001. (**c**) Representative 3D confocal images of ATII cells cultured in Matrigel in the absence or presence of Baf-A1 (10 nM) for 48 hrs. Spheres were stained for F-actin with Rhodamine-phalloidin (red) and DAPI (blue). Scale bars: 40μm.

**Supplementary Figure 3** Autophagy inhibition by *ATG5* depletion in alveolar epithelial cells induces EMT and invasion. (**a**) Protein expression of E-cadherin, Snail2, ATG12–ATG5, LC3 and p62/SQSTM1 in A549 cells transfected with control or *ATG5* siRNA. β-tubulin was used as a loading control. (**b**) Fold change in mRNA levels of *CDH1* (E-cadherin), *SNAI1* (Snail1), *SNAI2* (Snail2), *TWIST1*, *ZEB1* and *ZEB2* in A549 cells transfected with control or *ATG5* siRNA. GAPDH-normalised mRNA levels in control cells were used to set the baseline value at unity. Data are mean ± s.d. *n* = 3 samples per group. *** *P* < 0.001. (**c**) Immunofluorescence staining of p62/SQSTM1 (red) and E-cadherin (green) in A549 cells transfected with control or *ATG5* siRNA. Scale bar: 20 μm. (d) Transwell Matrigel invasion assays in control of *ATG5*-depleted ATII cells. Cells were stained with crystal violet. Data are mean ± s.d. *n* = 3. *** *P* < 0.001.

**Supplementary Figure 4** Autophagic activity altered by Rapamycin affects cellular plasticity of ATII cells. (**a**) Protein expression of p62/SQSTM1, phospho-mTOR (p-mTOR), mTOR, ATG12–ATG5 and LC3 with indicated treatments in ATII cells. β-tubulin was used as a loading control. (**b)** Fold change in mRNA levels of *CDH1* (E-cadherin), *VIM* (Vimentin) and *SNAI2* (Snail2) in ATII cells with indicated treatments. β-actin normalised mRNA levels in control cells were used to set the baseline value at unity. Data are mean ± s.d. *n* = 3 samples per group. * *P* < 0.05. ** *P* < 0.01. *** *P* < 0.001.

**Supplementary Figure 5** Autophagy inhibition induces EMT via p62/SQSTM1-NF-κB-Snail2 pathway in A549 cells. (**a**) Protein expression of ATG12–ATG5, p65/RELA and p62/SQSTM1 with indicated treatments in A549 cells. β-tubulin was used as a loading control. (**b)** Fold change in mRNA levels of *CDH1* (E-cadherin) or *SNAI2* (Snail2) in A549 cells with indicated treatments. GAPDH-normalised mRNA levels in control cells were used to set the baseline value at unity. Data are mean ± s.d. *n* = 3 samples per group. *** *P* < 0.001.

**Supplementary Figure 6** ATII cells undergoing autophagy inhibition-induced EMT induce fibroblast activation via Snail2-regulated paracrine signalling. Fold change in mRNA levels of *COL1A1, COL3A1, FN1* and *ACTA2* in ATII cells transfected with the indicated siRNA. β-actin-normalised mRNA levels in control. Data are mean ± s.d. *n* = 3 samples per group. ** *P* < 0.01. *** *P* < 0.001.
