# Supplementary figures and images for: Autophagy inhibition-mediated epithelial–mesenchymal transition augments local myofibroblast differentiation in pulmonary fibrosis
Source: Cell Death Dis. 2019 Aug 7;10(8):591. doi: 10.1038/s41419-019-1820-x (PMC6685977; doi:10.1038/s41419-019-1820-x)

## Supplementary Figure 1

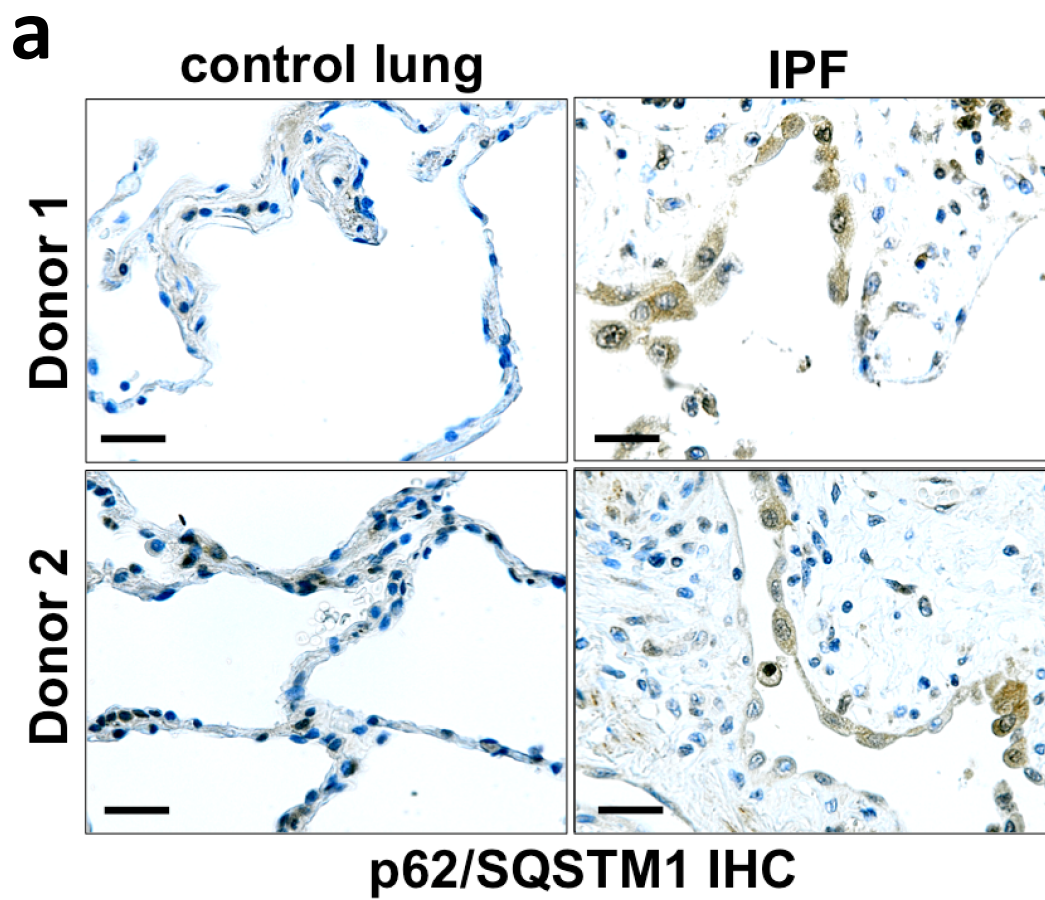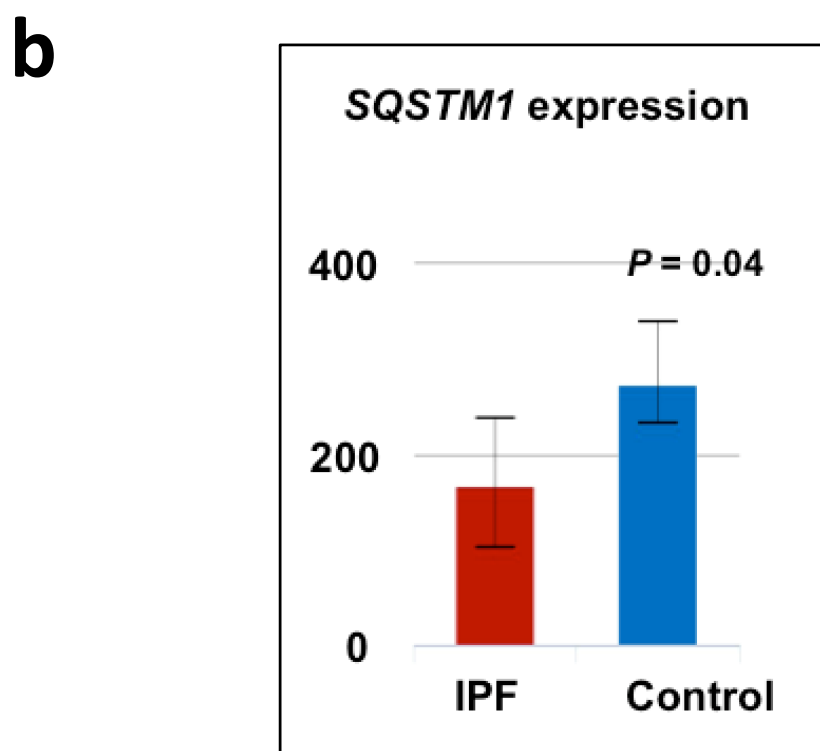

Supplement: Supplementary file 2 — Supplementary Figure S1. [file 41419_2019_1820_MOESM2_ESM.pdf]

## Supplementary Figure 2

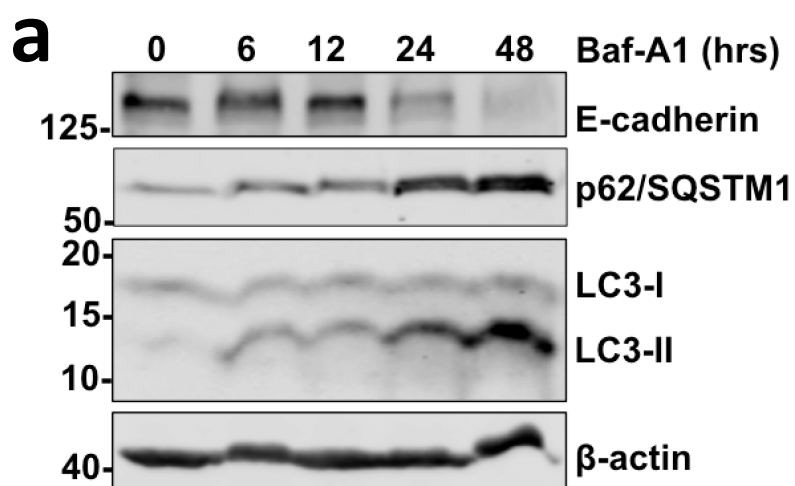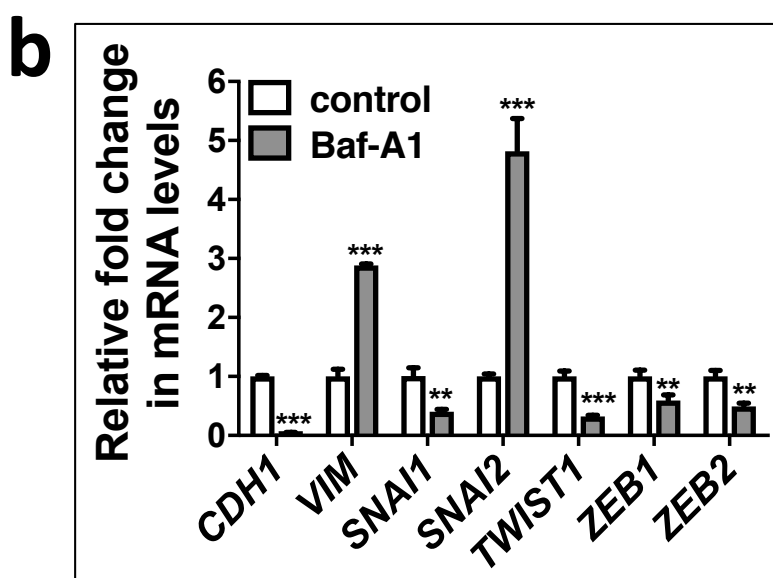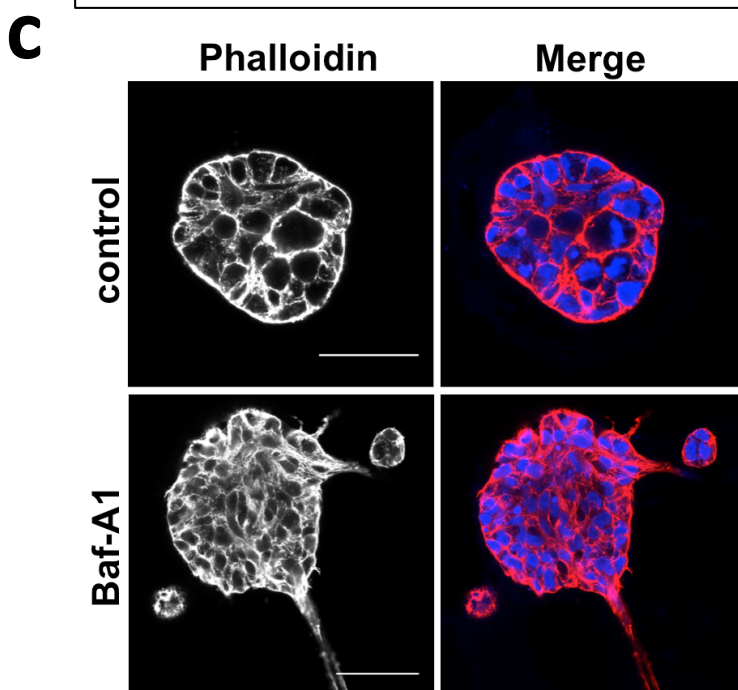

Supplement: Supplementary file 3 — Supplementary Figure S2. [file 41419_2019_1820_MOESM3_ESM.pdf]

## Supplementary Figure 3

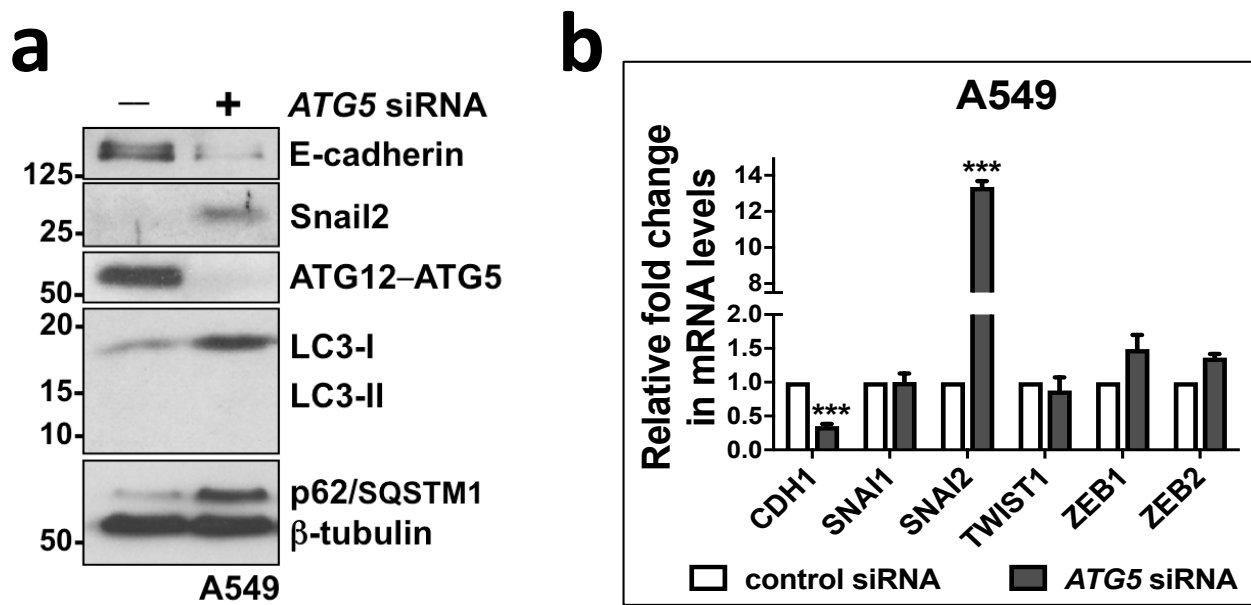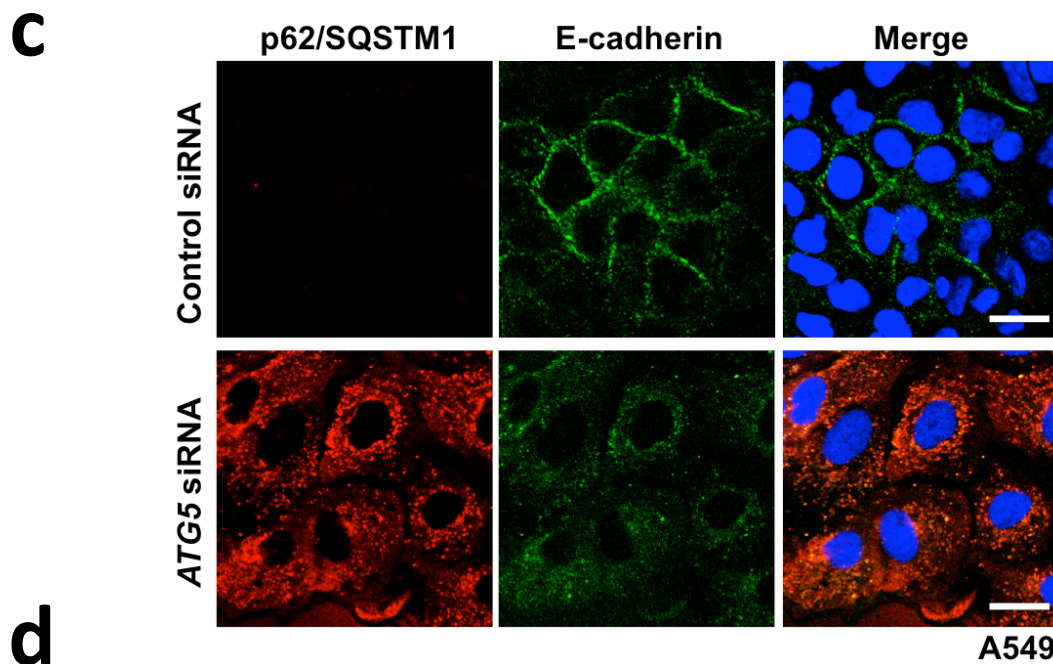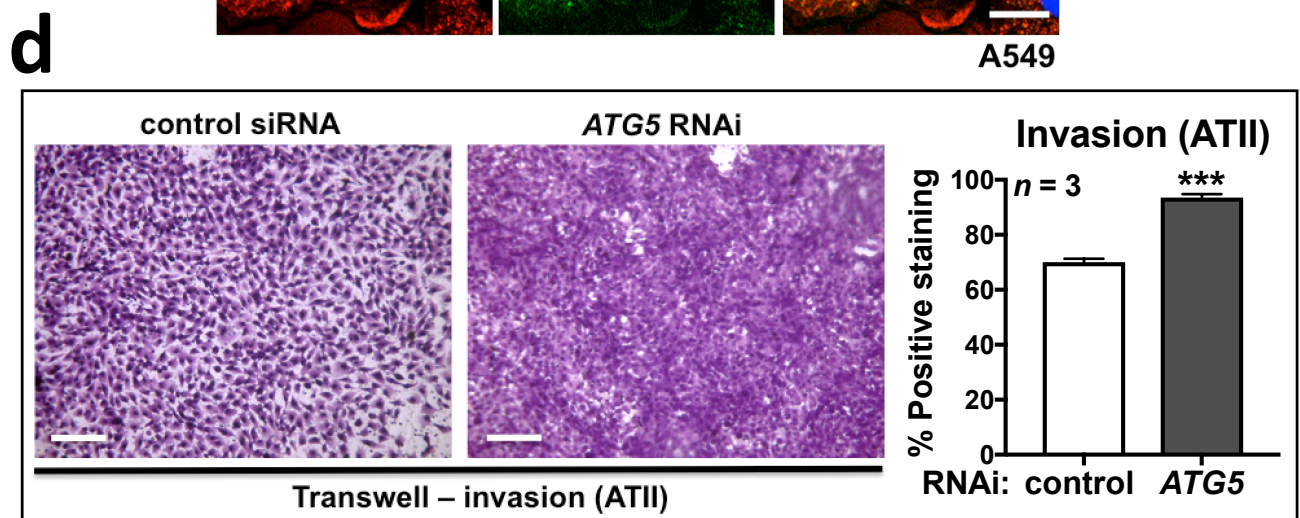

Supplement: Supplementary file 4 — Supplementary Figure S3. [file 41419_2019_1820_MOESM4_ESM.pdf]

## Supplementary Figure 4

**a**

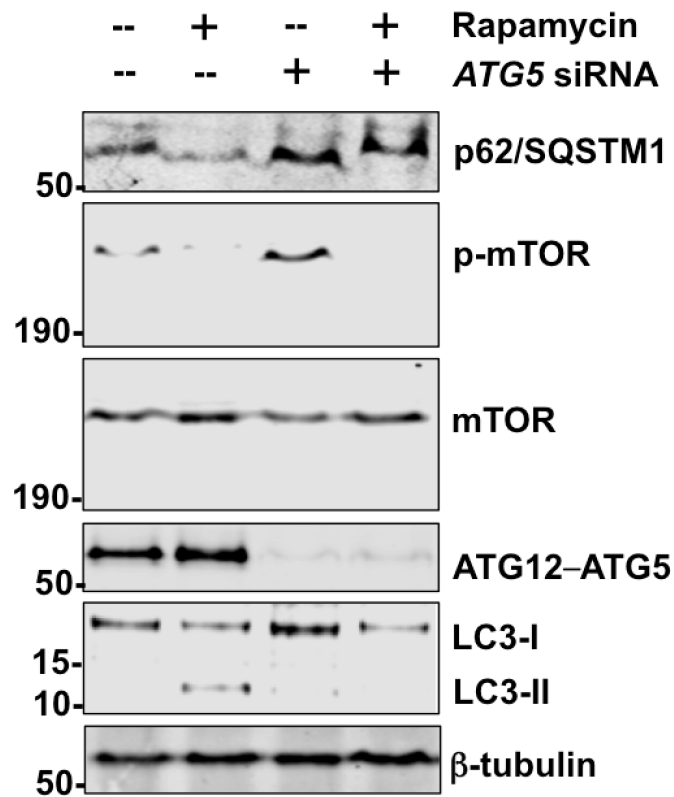

**b**

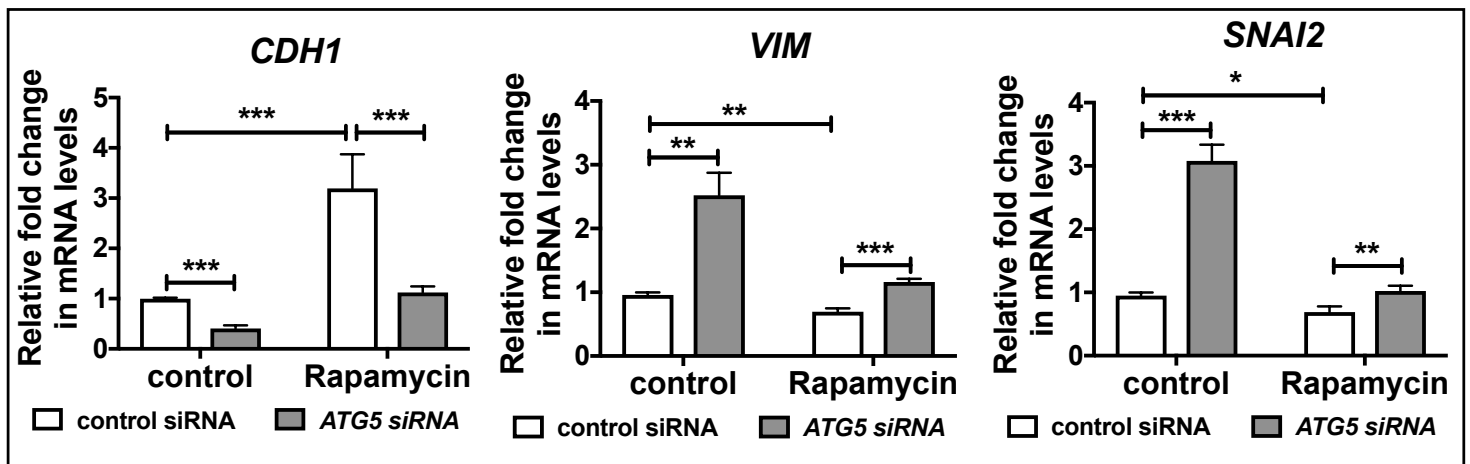

Supplement: Supplementary file 5 — Supplementary Figure S4. [file 41419_2019_1820_MOESM5_ESM.pdf]

## Supplementary Figure 5

**a**

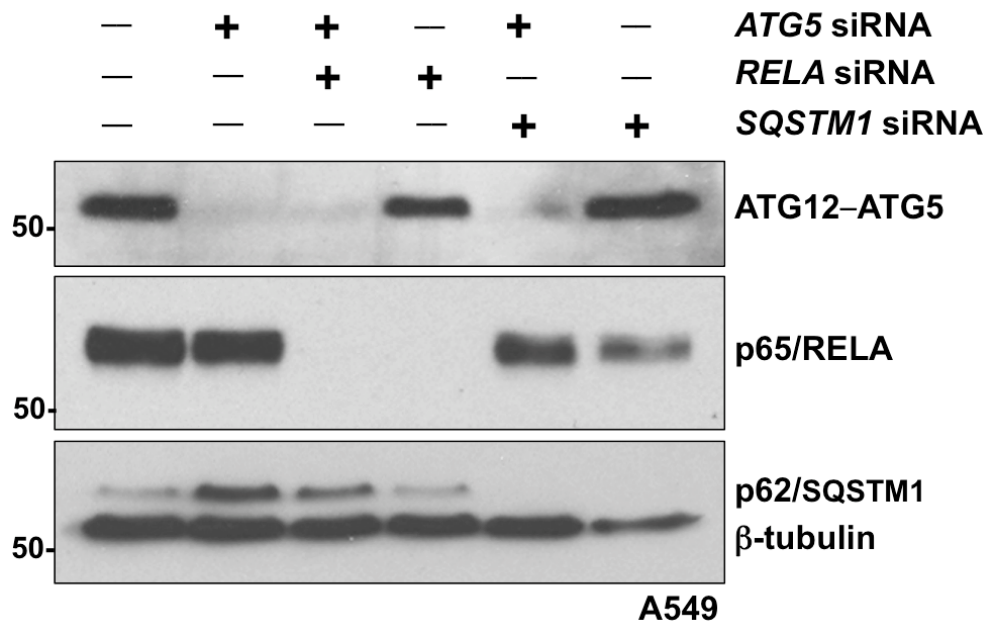

**b**

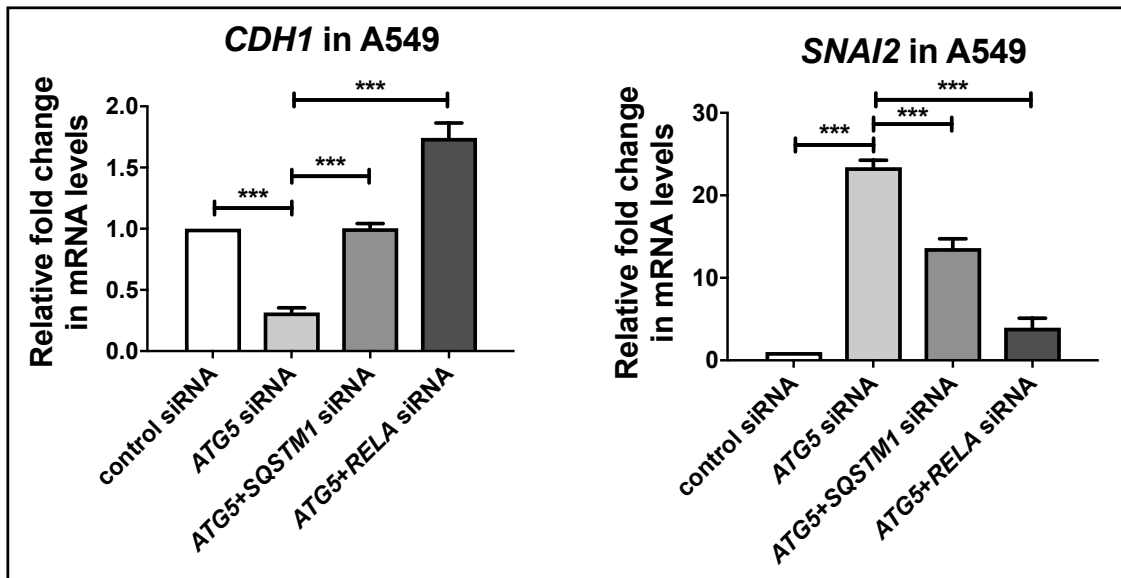

Supplement: Supplementary file 6 — Supplementary Figure S5. [file 41419_2019_1820_MOESM6_ESM.pdf]

Supplementary Figure 6

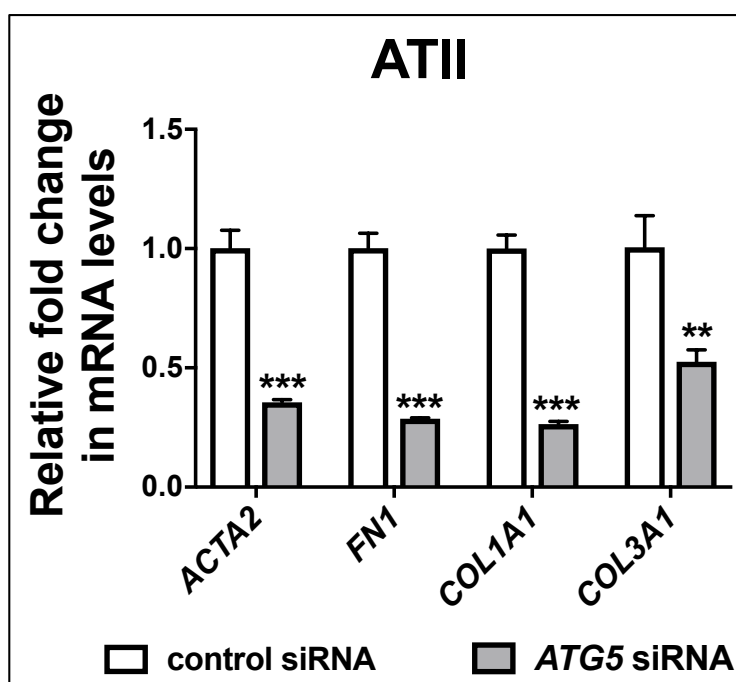

Supplement: Supplementary file 7 — Supplementary Figure S6. [file 41419_2019_1820_MOESM7_ESM.pdf]
